# The Impact of Acute and Chronic Stress and Cannabinoid Extract Injection on Orthodontic Tooth Movement: An Experimental Rat Model Study

**DOI:** 10.1155/ijod/3913540

**Published:** 2025-12-11

**Authors:** Amin Golshah, Mohammad Moslem Imani, Fatemeh Azizi, Farzad Rezaei, Navid Rezaei, Zohre Farhangian, Fatemeh Ghorbani, Nafiseh Nikkerdar

**Affiliations:** ^1^ Department of Orthodontics, School of Dentistry, Kermanshah University of Medical Sciences, Kermanshah, Iran, kums.ac.ir; ^2^ Department of Oral and Maxillofacial Surgery, School of Dentistry, Kermanshah University of Medical Sciences, Kermanshah, Iran, kums.ac.ir; ^3^ Department Operative of Dentistry, Faculty of Dentistry, Kermanshah University of Medical Sciences, Kermanshah, Iran, kums.ac.ir; ^4^ Student Research Committee, School of Dentistry, Kermanshah University of Medical Sciences, Kermanshah, Iran, kums.ac.ir; ^5^ Department of Oral and Maxillofacial Radiology, School of Dentistry, Kermanshah University of Medical Sciences, Kermanshah, Iran, kums.ac.ir

**Keywords:** acute stress, cannabinoid extract, chronic stress, orthodontic tooth movement

## Abstract

**Background and Objectives:**

Knowledge about the effects of medications and supplements on orthodontic tooth movement (OTM) is imperative for orthodontists. This study aimed to assess the effects of acute and chronic stress and cannabinoid extract (CE; marijuana) injection on OTM in rats.

**Materials and Methods:**

In this animal experimental study, 220 male Wistar rats were randomly assigned to 22 subgroups (*n* = 10) in two main groups of acute and chronic stress. The rats in the acute group were evaluated over a 21‐day period in the following 11 subgroups (1) no orthodontic treatment/no medication (2), orthodontic treatment/no medication (3), no orthodontic treatment/intraperitoneal CE injection (4), orthodontic treatment/CE injection (5), no orthodontic treatment/saline injection (6), orthodontic treatment/saline injection (7), no orthodontic treatment/no medication under stressful conditions (8), orthodontic treatment/no medication under stressful conditions (9), no orthodontic treatment/CE injection under stressful conditions (10), orthodontic treatment/CE injection under stressful conditions (11), no orthodontic treatment/saline injection under stressful conditions. The rats in the chronic group were studied over a 40‐day period in the following 11 subgroups with the same subgroups of the acute group. All rats were then sacrificed, their maxilla was resected, and OTM, the mean number of blood vessels, osteoclasts, and Howship’s lacunae, bone volume/total volume (BV/TV) ratio, and bone mineral density (BMD) were assessed.

**Results:**

OTM was significantly greater under chronic stress (0.23 ± 0.19 mm) than acute stress (0.21 ± 0.16 mm, *p*  < 0.001), and in rats receiving CE injection (0.29 ± 0.19 mm) compared with controls (0.20 ± 0.17 mm, *p*  < 0.001). The mean number of Howship’s lacunae (10.74 ± 5.27 vs. 8.52 ± 6.31, *p*  < 0.001), osteoclasts (14.69 ± 7.53 vs. 11.06 ± 8.45, *p*  < 0.001), and blood vessels (12.45 ± 3.19 vs. 10.81 ± 2.69, *p*  < 0.001) were also significantly higher in stressed rats receiving CE. BV/TV and BMD were significantly lower in CE‐treated stressed rats (BV/TV: 17.56% ± 2.20% vs. 27.79% ± 5.68%, *p*  < 0.001; BMD: 1.76 ± 0.39 vs. 2.54 ± 0.46 g/cm^3^, *p*  < 0.001) compared with controls. All parameters were further enhanced by orthodontic treatment.

**Conclusion:**

Daily CE injection combined with acute and chronic stress significantly enhances OTM in rats, accompanied by increased osteoclastic activity, vascularization, and decreased bone density. Quantitative data and statistical significance provide robust evidence of these effects.

## 1. Introduction

Orthodontic treatment is highly popular among the general population due to enhanced public knowledge about the significance of dental esthetics [1]. Orthodontic tooth movement (OTM) is defined as the correction of tooth position by the application of orthodontic forces. This process includes biomechanical adaptation and remodeling of the alveolar bone and the periodontium [2, 3]. Remodeling of the periodontium and subsequent OTM is a complex biological process that includes bone resorption at the pressure side by the activity of osteoclasts and bone neogenesis at the tension side by the activity of osteoblasts [4]. The proliferation rate of osteoclasts can be used as an important index for the determination of OTM. When teeth reach their desired position, the alveolar bone and the periodontal ligament (PDL) regain their normal structure [5]. Orthodontic treatment often lasts 1–2 years. Sufficient knowledge about the effects of medications, vitamins, supplements, and illicit drugs on OTM is imperative for orthodontists. These substances can reach the periodontium under orthodontic forces through the blood circulation and accelerate or decelerate the course of orthodontic treatment [6]. Nonetheless, information about the interactions of medications with OTM is limited, and the risk of adverse effects always exists.

Stress is the psychological response of the human body to different physical and emotional stimuli, resulting in the release of neuroendocrine hormones with significant effects on bone [7–10]. Osteoblasts and osteoclasts are affected by the glucocorticoid and adrenergic receptors. Cortisol is among the hormones released in the human body under stress, which can affect the performance of osteoblasts and osteoclasts and has significant impacts on the osteoclastogenesis process in response to orthodontic force application [11, 12]. The role of stress in osteogenesis has been the topic of many investigations, and it has been demonstrated that unpredictable stress elicits a severe inflammatory response and leads to subsequent bone loss [13–16].

Cannabinoids have been the topic of many investigations due to their therapeutic properties. Marijuana contains high amounts of cannabinoids, which are bioactive molecules that exert their effects mainly through the cannabinoid receptors [17, 18]. The dominant cannabinoid present in the composition of marijuana can activate the cannabinoid receptors in the human body. In addition to their known effects on the central nervous system, cannabinoids can affect the activity of osteoclasts, bone mass, and bone regeneration. Cannabinoids have two receptors of CB1 and CB2. CB2 is the main cannabinoid receptor paired with G protein that regulates the proliferation, differentiation, and maturation of osteoclasts, osteoblasts, and osteocytes [19–23]. CB2 plays an important role in bone homeostasis [2]. A number of studies have evaluated the effects of cannabinoids on bone homeostasis. Evidence shows that CB2 stimulates osteoclastogenesis and bone loss [24–29]. In the process of bone remodeling, administration of high doses of marijuana on a daily basis can decrease the bone density and increase the risk of bone fracture and osteoporosis [30]. Computed tomography (CT) and histomorphometric studies on bone formation and resorption indicated a lower number of osteoclasts and less bone resorption in CB1 receptor‐deficient rats. Evidence shows that pharmaceutical inhibition of CB2 receptor inhibits osteoclastogenesis, and results in bone loss in adults [31, 32]. It should be noted that different doses of cannabinoids may have variable effects. It has been demonstrated that lower doses of cannabinoids can stimulate bone resorption; however, using very high doses of cannabinoids inhibits both osteoblasts and osteoclasts [33–35]. Moreover, it should be noted that similar doses of cannabinoids may have widely variable blood concentrations in different individuals, which can lead to indefinite results in patients under treatment with a certain dose [33]. Despite the increasing use of hashish and cannabinoids worldwide, the effects of cannabinoid extract (CE) on CB2 receptors, OTM, and alveolar bone remodeling are unclear and need further investigations [2]. Considering the growing popularity of marijuana (cannabinoid) and exposure to different stressful conditions, this study aimed to assess the biological and histological effects of intraperitoneal injection of CE and stress on OTM in rats.

## 2. Materials and Methods

### 2.1. Animal Model and Study Groups

This animal study was ethically approved by the Ethics Committee of Kermanshah University of Medical Sciences (IR.KUMS.REC.1399.120) and was conducted in accordance with the ARRIVE guidelines [36]. Also, the study adhered to the guidelines for the care and use of laboratory animals. This experimental study evaluated 220 male Wistar rats, weighing 200–250 g, that were purchased from the Kermanshah University of Medical Sciences. For the purpose of acclimation, the rats were kept in transparent plastic cages at 24–25°C temperature with 55% humidity, and a 12‐h light/12‐h dark cycle for 1 week. The rats received soft food during the study period to minimize the risk of orthodontic appliance debonding [37]. Orthodontic appliances were installed for all rats as explained in previous studies [38], except those in subgroups that did not undergo orthodontic treatment.

The rats were randomly assigned to 22 subgroups (*n* = 10) in two main groups of acute and chronic stress. The rats in the acute stress group were evaluated over a 21‐day period in the following subgroups:

1. The rats in this subgroup did not receive any medication and did not undergo orthodontic treatment.
2. The rats in this subgroup did not receive any medication, but they underwent orthodontic treatment.
3. The rats in this subgroup did not undergo orthodontic treatment but received intraperitoneal injections of 5 mg/kg body weight CE on a daily basis.
4. The rats in this subgroup underwent orthodontic treatment and received intraperitoneal injections of 5 mg/kg CE on a daily basis.
5. The rats in this subgroup did not undergo orthodontic treatment but received 1 mL/kg body weight saline on a daily basis.
6. The rats in this subgroup underwent orthodontic treatment and received 1 mL/kg body weight saline on a daily basis.
7. The rats in this subgroup were subjected to stressful conditions but did not receive any medication and did not undergo orthodontic treatment.
8. The rats in this subgroup were subjected to stressful conditions, underwent orthodontic treatment but did not receive any medication.
9. The rats in this subgroup were subjected to stressful conditions. They did not undergo orthodontic treatment but received intraperitoneal injection of 5 mg/kg body weight CE on a daily basis.
10. The rats in this subgroup were subjected to stressful conditions. They underwent orthodontic treatment and also received intraperitoneal injections of 5 mg/kg body weight CE on a daily basis.
11. The rats in this subgroup were subjected to stressful conditions. They did not undergo orthodontic treatment but received 1 mL/kg body weight saline on a daily basis.

**Figure 1:**
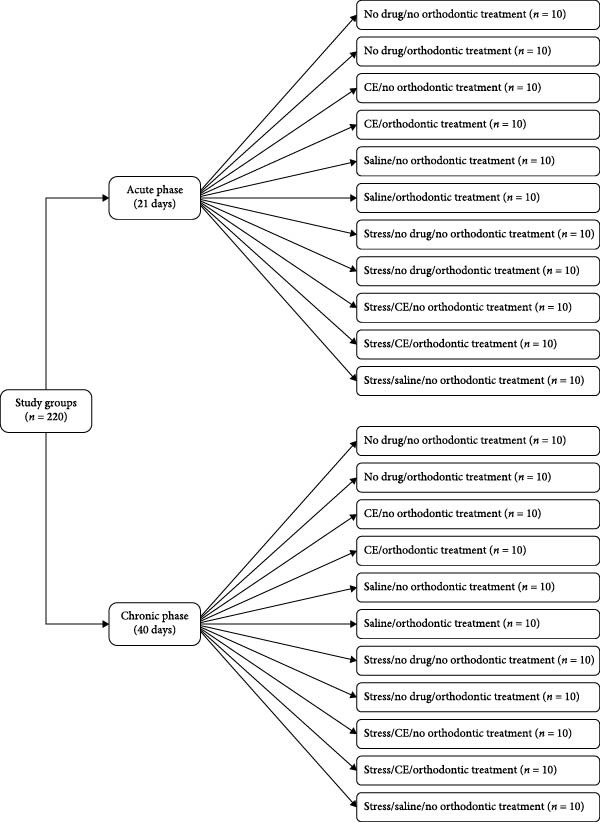
Flowchart of the study groups.

The rats in the chronic stress group were evaluated over a 40‐day period in the following subgroups:

1. The rats in this subgroup did not receive any medication and did not undergo orthodontic treatment.
2. The rats in this subgroup did not receive any medication, but they underwent orthodontic treatment.
3. The rats in this subgroup did not undergo orthodontic treatment but received intraperitoneal injections of 5 mg/kg body weight CE on a daily basis.
4. The rats in this subgroup underwent orthodontic treatment and received intraperitoneal injections of 5 mg/kg CE on a daily basis.
5. The rats in this subgroup did not undergo orthodontic treatment but received 1 mL/kg body weight saline on a daily basis.
6. The rats in this subgroup underwent orthodontic treatment and received 1 mL/kg body weight saline on a daily basis.
7. The rats in this subgroup were subjected to stressful conditions, but did not receive any medication and did not undergo orthodontic treatment.
8. The rats in this subgroup were subjected to stressful conditions, underwent orthodontic treatment but did not receive any medication.
9. The rats in this subgroup were subjected to stressful conditions. They did not undergo orthodontic treatment but received intraperitoneal injections of 5 mg/kg body weight CE on a daily basis.
10. The rats in this subgroup were subjected to stressful conditions. They underwent orthodontic treatment and also received intraperitoneal injections of 5 mg/kg body weight CE on a daily basis.
11. The rats in this subgroup were subjected to stressful conditions. They did not undergo orthodontic treatment but received 1 mL/kg body weight saline on a daily basis (Figure 1).

### 2.2. Orthodontic Appliance Installation

**Figure 2:**
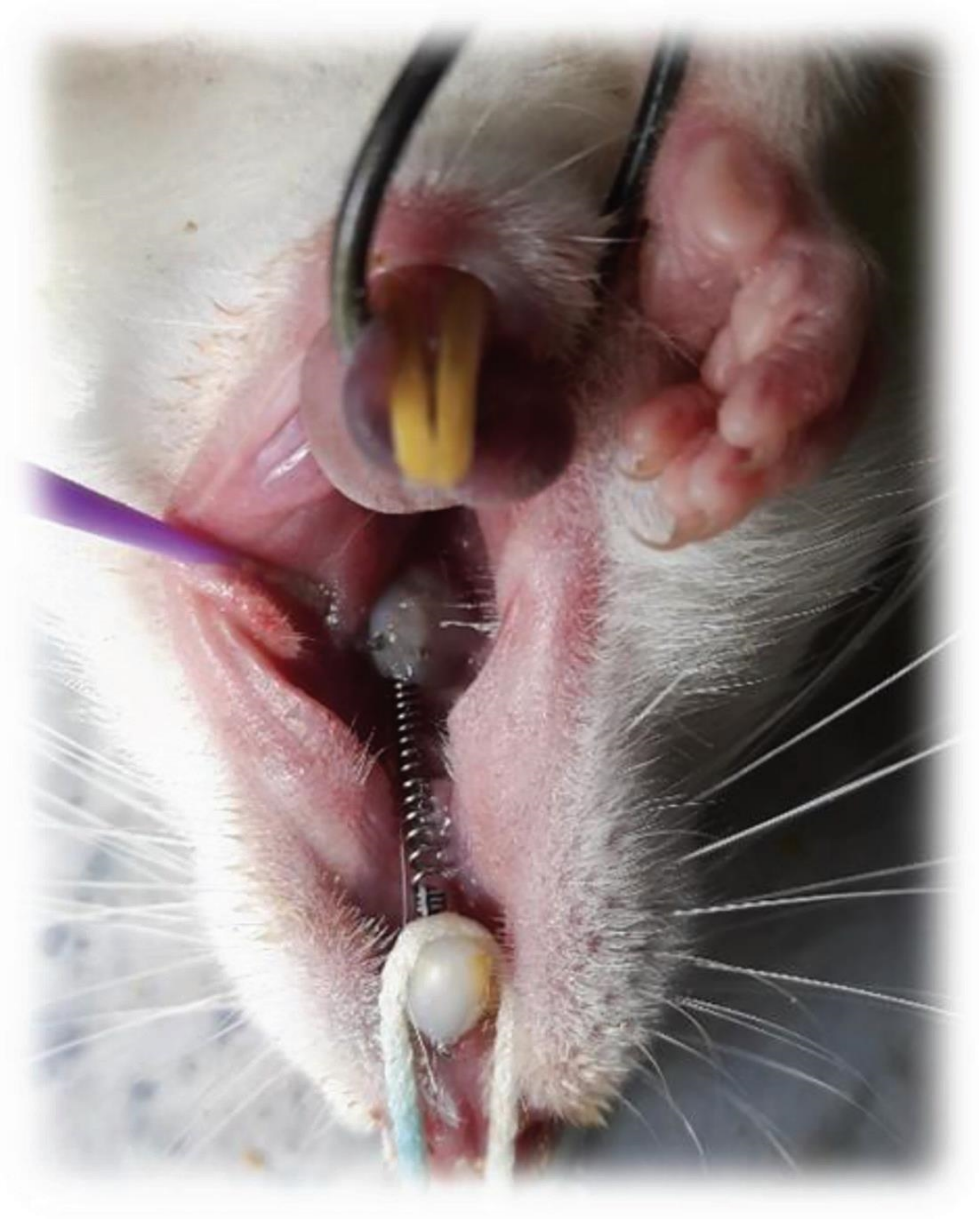
NiTi coil spring placed between the first molar and central incisor of a rat.

To induce general anesthesia, the rats received 10% ketamine hydrochloride (50 mg/kg; Alfasan, Worden, the Netherlands) and 2% xylazine (2 mg/kg; Alfasan, Worden, the Netherlands) intravenously. After anesthesia induction, the vital signs of the rats were monitored, and they were rolled over every couple of minutes to prevent pulmonary edema. The room temperature was adjusted as well. Nickel–titanium coil springs (6 mm; G & H Franklin) were used to induce OTM. Stainless‐steel wires were connected to the first molars and central incisors of each rat. The teeth were etched with 37% phosphoric acid (Vivadent, USA) for 30 s, rinsed for 10 s, and air‐dried for 15 s. Single Bond (3M ESPE, St. Paul, MN, USA) was applied on the tooth surfaces and cured with an LED curing unit (Woodpecker, Muenster, Germany) with a light intensity of 150 mW/cm^2^ for 10 s. The orthodontic appliance was secured by the application of Transbond XT light‐cure composite resin (3M ESPE, St. Paul, MN, USA). The coil spring exerted 50 g force [39] (Figure 2) as measured by a force‐meter. The mandibular central incisors were also shortened to prevent potential damage to the orthodontic appliance. All rats were monitored on a daily basis to ensure correct positioning of the coil spring. In case of its displacement, the rat would be excluded from the study and replaced [40].

### 2.3. Exposure to Stress

Acute stress was induced by 1‐h daily restraint for three consecutive days (Days 11, 12, and 13 after appliance placement), while chronic stress was induced by 1‐h daily restraint for 5 days/week over a total period of 40 days, following the protocol described by Gameiro et al. [41]. The restraint involved placing the rats in a plastic device adjustable in size according to the rat’s weight for 1‐h. The interior of the tube was adjustable for each rat, with a mobile internal wall, which was secured in place with Velcro straps. The tube also had a 1‐cm breathing hole at one end [42].

### 2.4. Cannabinoid Extraction

The cannabis plant was obtained from the Drug Control Headquarters of Iran and subsequently verified by an expert at the Biosystematics Center of Kermanshah University of Medical Sciences. The plant was powdered, and 50 g of the powder was added to 500 mL of 70% ethanol (Merck, Germany) in a percolator apparatus (Arian Equip, Iran) for 72 h. Next, the percolator valve was opened to collect the extract. It should be noted that ethanol was added from the top through the detonator to ensure that the solution exiting the percolator is completely colorless. The solvent was subsequently evaporated in a rotary evaporator (IKA, Germany) operating at 50 rpm at 45°C temperature, yielding a viscous honey‐like substance, which was then dried in a desiccator (Pyrex, Germany) connected to a vacuum pump and subsequently transferred to a dark‐colored presterilized glass container. The CE at 5 mg/kg concentration was administered intraperitoneally on a daily basis [43, 44].

### 2.5. Quantification of OTM

The distance between the distal surface of the first molar and the mesial surface of the second molar was measured at baseline (Day 1) and also after 21 and 40 days by one single operator blinded to the group allocation of the rats, using AB Viewer 14 software. The measurements were repeated in triplicate, and the mean of the three values was calculated and recorded. Dental impressions were also made using polyvinyl siloxane impression material (Express; 3M ESPE, St. Paul, MN, USA) at 1, 21, and 40 days. The impressions were allowed 4 min to set. Next, the tray was removed, and the impressions were poured with dental stone (Elite Rock Dental Stone; Zhermack, Italy). The cast remained in the impression for 24 h before removal [5].

### 2.6. Histological and Immunohistochemical Analyses

Specimen preparation: The rats were euthanized by chloroform inhalation in a saturated desiccator after 21 and 40 days. The maxilla was surgically resected for histological analysis. The specimens were first fixed in 10% formaldehyde and then decalcified by immersion in 10% formic acid for 48 h (Sigma–Aldrich, St. Louis, MO, USA). Decalcification was performed by immersion in 12.5% ethylene diamine tetra‐acetic acid, followed by a 10‐week fixation period. The decalcifying solution was agitated 10 times/day and refreshed twice a week until complete decalcification occurred. Subsequently, all specimens were dehydrated by using ethanol and embedded in paraffin blocks. Next, 5‐μm thick parasagittal sections were made by a microtome (Leica, Wetzlar, Germany) [45]. Histological analyses were performed by one examiner (A.H.Y) blinded to the group allocation of the specimens. Tissue specimens were hematoxylin and eosin stained and were examined under a light microscope (Eclipse E400, Nikon, Japan) at ×100 magnification by a qualified pathologist blinded to the group allocations. The number of Howship’s lacunae, blood vessels, and osteoclasts was counted in a 0.01‐mm^2^ designated area. Each parameter was counted three times, and the mean of the three values was calculated and reported [5].

### 2.7. Micro‐CT Assessment

The extracted maxillae also underwent micro‐CT in an X‐ray micro‐CT scanner (LOTUS inVivo, Behin Negareh Co., Tehran, Iran). LOTUS‐inVivo has a cone beam micro‐focus X‐ray source and a flat panel detector with the exposure settings of 60 kV tube potential and 100 *μ*A tube current and a frame exposure time of 1 s by 4 magnification and the total scanning time of 49 min to obtain the best possible image quality. The reconstructed images had a 20‐µm slice thickness. All settings were controlled by LOTUS‐inVivo‐ACQ software. The acquired 3D data were reconstructed using LOTUS inVivo‐REC by a standard Feldkamp, Davis, Kress (FDK) algorithm. The bone volume/total volume (BV/TV) ratio and bone mineral density (BMD) were also recorded. To calculate the BV/TV ratio, the molar roots were first segmented. Next, all “holes” (corresponding to the root canals) were filled by applying a dilation filter to increase the root size. The originally sized roots were then subtracted from the enlarged roots to obtain the volume of interest (VOI). To calculate the BV/TV within the VOI, the amount of bone within the VOI was quantified. The micro‐CT‐HA phantom (QRM brand) was used for the calculation of BMD. In the micro‐CT‐HA phantom, the attenuation coefficient of each material and also the relationship between the density and the attenuation coefficient were calculated. Next, the BMD of the samples was calculated based on the relationship of the density and the attenuation coefficient for the phantom sample [46].

### 2.8. Statistical Analysis

Data were analyzed descriptively and inferentially. The measures of central dispersion were reported descriptively. Inferentially, first, the normality of data distribution was analyzed by the Kolmogorov–Smirnov test. Next, comparisons were made by four‐way ANOVA followed by pairwise comparisons with the Tukey’s post hoc test. All statistical analyses were conducted using IBM SPSS Statistics for Windows, version 26.0. (IBM Corp. Released 2019. IBM Corp., Armonk, NY, USA) at 0.05 level of significance.

## 3. Results

### 3.1. Effects of Time, Stress, Orthodontic Treatment, and CE Injection on OTM

Four‐way ANOVA was applied to assess the effects of time, stress, orthodontic treatment, and CE injection on OTM. As shown in Table 1, time had a significant effect on OTM (*p*  < 0.001), such that the mean OTM at 40 days (chronic period) was significantly greater than that at 21 days (acute period). Stress had a significant effect on OTM as well (*p*  < 0.001), such that the mean OTM was significantly greater under stressful conditions, compared with the absence of stress. Orthodontic treatment also had a significant effect on OTM (*p*  < 0.001), such that the mean OTM in the subgroups under orthodontic treatment was significantly greater than that in the subgroups without orthodontic treatment. CE injection had a significant effect on OTM as well (*p*  < 0.001), such that the mean OTM in the subgroups that received CE injection was significantly greater than that in other subgroups.

**Table 1:** Results of four‐way ANOVA regarding the effects of time, stress, orthodontic treatment, and CE injection on OTM.

Experimental factors		Distance 0 (mm)		Distance 1 (mm)		Movement		
		Mean	SD	Mean	SD	Mean	SD	*p*‐Value^†^
Time	21 days	0.20	0.01	0.41	0.16	0.21^a^	0.16	**<0.001**
	40 days	0.20	0.01	0.44	0.19	0.23^b^	0.19	—

Stress	No	0.20	0.01	0.40	0.17	0.20^a^	0.17	**<0.001**
	Yes	0.20	0.01	0.45	0.18	0.24^b^	0.18	—

Orthodontics	No	0.20	0.01	0.28	0.04	0.07^a^	0.04	**<0.001**
	Yes	0.20	0.01	0.60	0.09	0.40^b^	0.09	—

Cannabinoid extract	No	0.20	0.01	0.40	0.17	0.20^b^	0.17	**<0.001**
	Cannabinoid	0.20	0.01	0.49	0.19	0.29^c^	0.19	—
	Saline	0.20	0.01	0.36	0.13	0.16^a^	0.13	—

*Note:* Significant values are shown in bold. Means with same superscript letters are not significantly different (*p* > 0.05).

Abbreviation: SD, standard deviation.

^†^Four way ANOVA followed by Tukey’s test was used.

Figure 3 shows the micro‐CT images of OTM in some subgroups.

Figure 3Micro‐CT analysis of OTM after 21 and 40 days: (A) orthodontic treatment/CE injection/stress (chronic); (B) orthodontic treatment/CE injection/stress (acute); (C) orthodontic treatment/stress (chronic); (D) orthodontic treatment/stress (acute); (E) no orthodontic treatment/CE injection/stress (chronic); (F) no orthodontic treatment/CE injection/stress (acute); (G) no orthodontic treatment/no drug (chronic); (H) no orthodontic treatment/no drug (acute).

**Figure:**
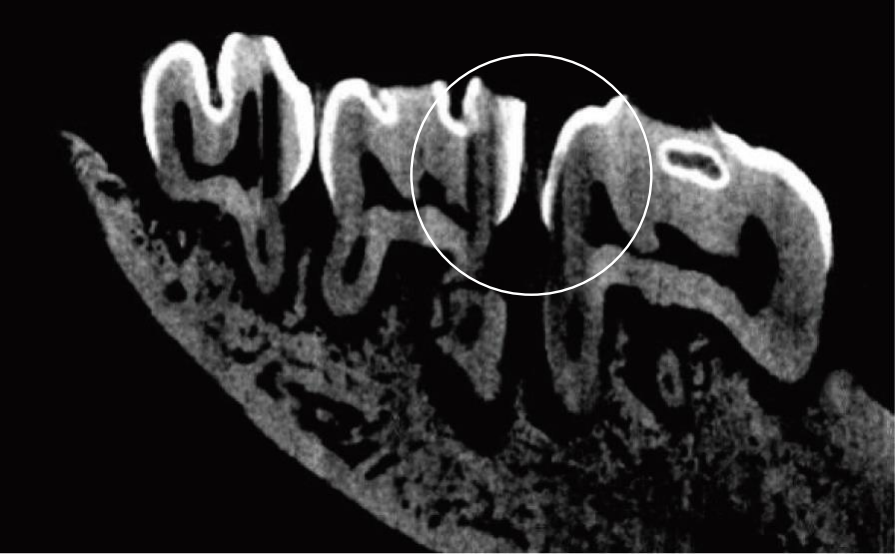
(A)

**Figure:**
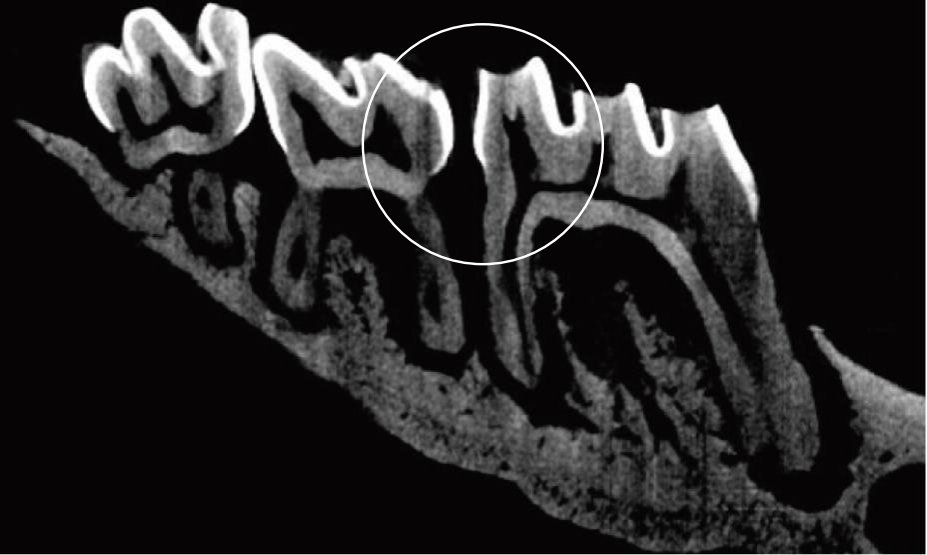
(B)

**Figure:**
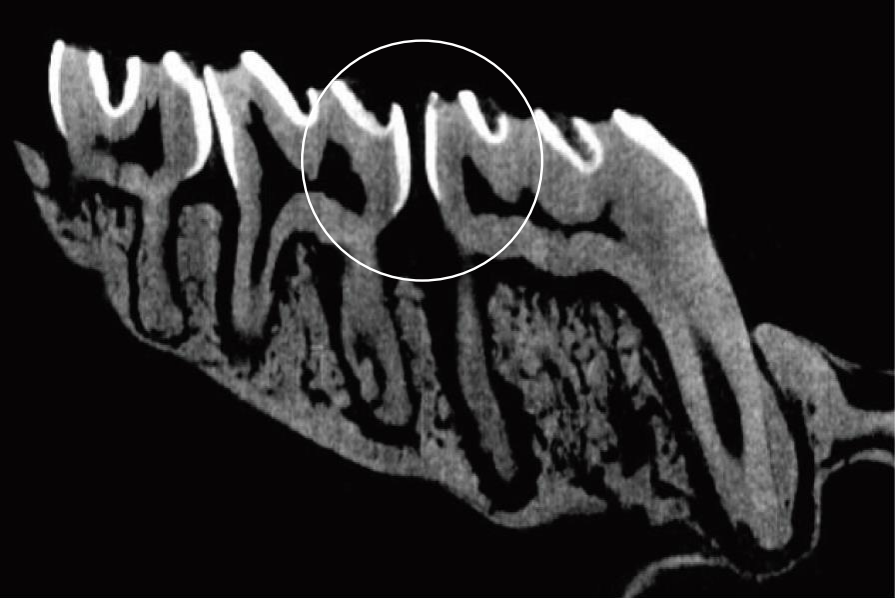
(C)

**Figure:**
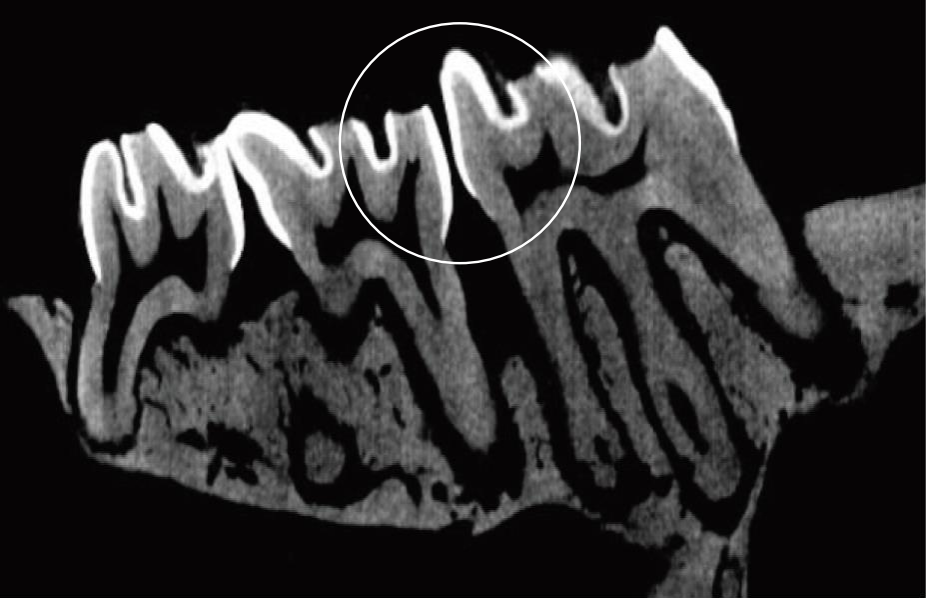
(D)

**Figure:**
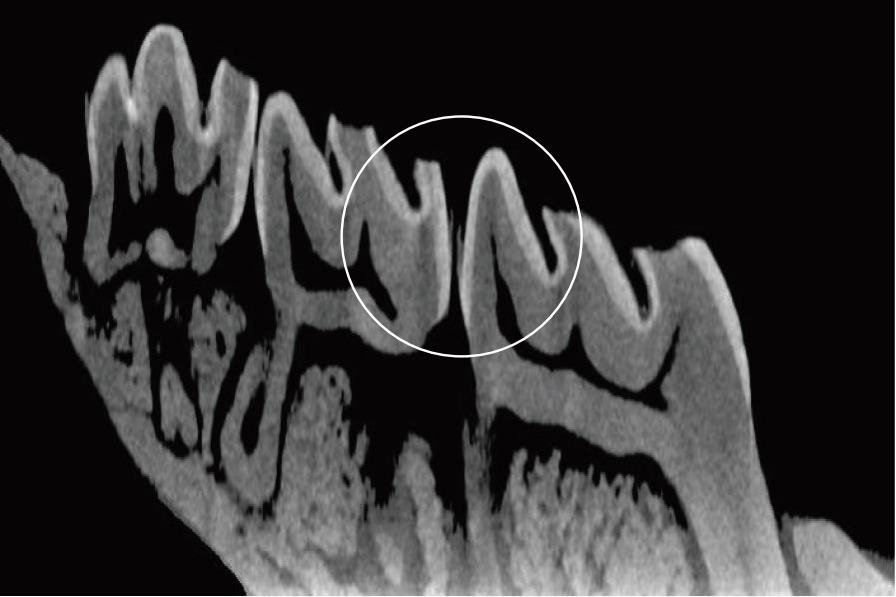
(E)

**Figure:**
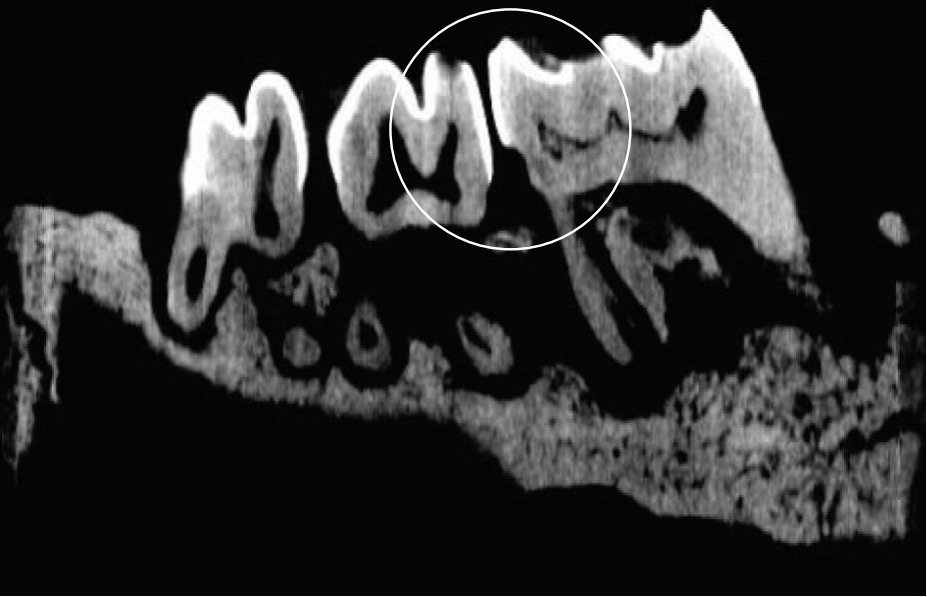
(F)

**Figure:**
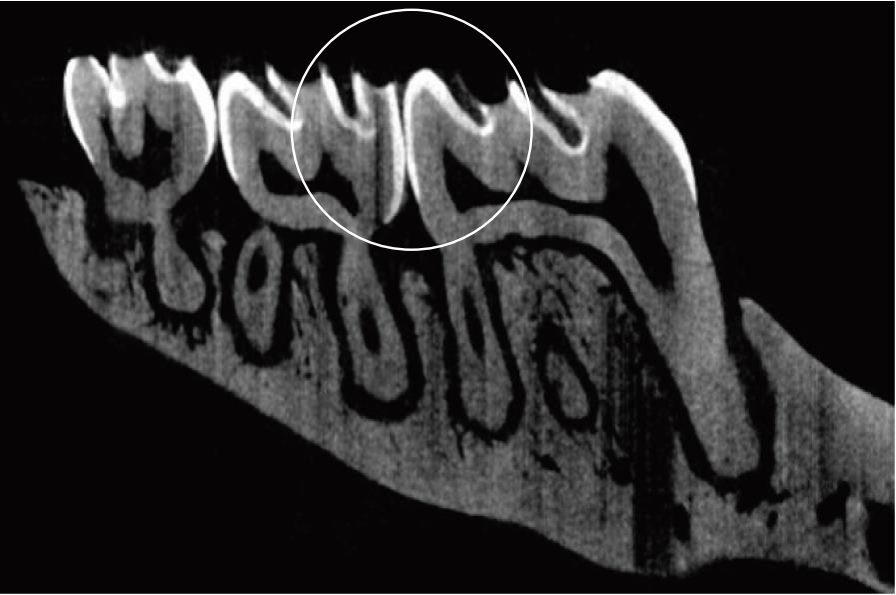
(G)

**Figure:**
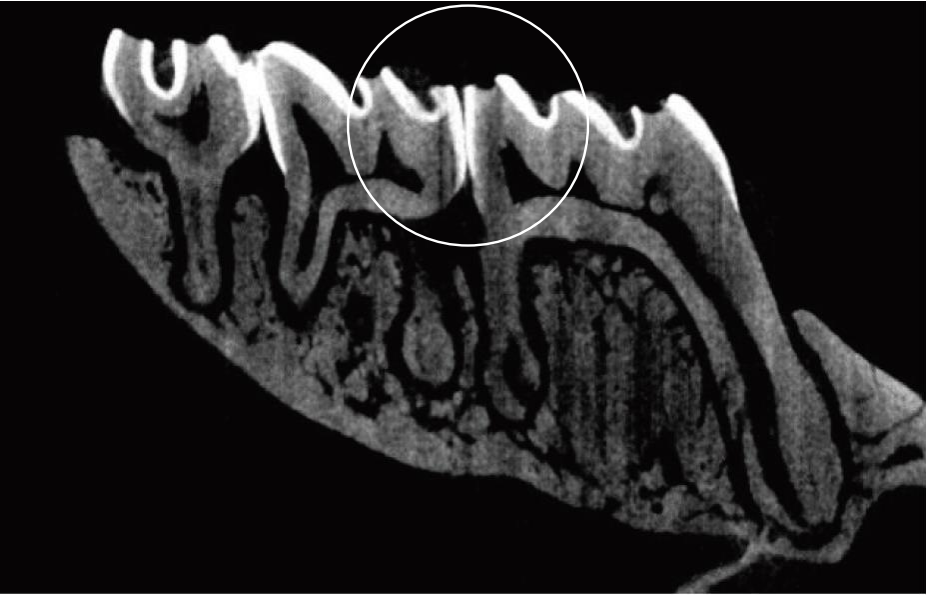
(H)

### 3.2. Histological Analysis

Figure 4 shows the periodontal tissue light‐microscopic findings in the study groups. As shown, the number of blood vessels, osteoclasts, and Howship’s lacunae was significantly higher in rats subjected to stress and CE injection for a 40‐day period, compared with others.

Figure 4H&E staining. Blood vessels and osteoclasts in different subgroups: (A) orthodontic treatment/CE injection/stress (chronic); (B) orthodontic treatment/CE injection/stress (acute); (C) no orthodontic treatment/CE injection/stress (chronic); (D) no orthodontic treatment/CE injection/stress (acute).

**Figure:**
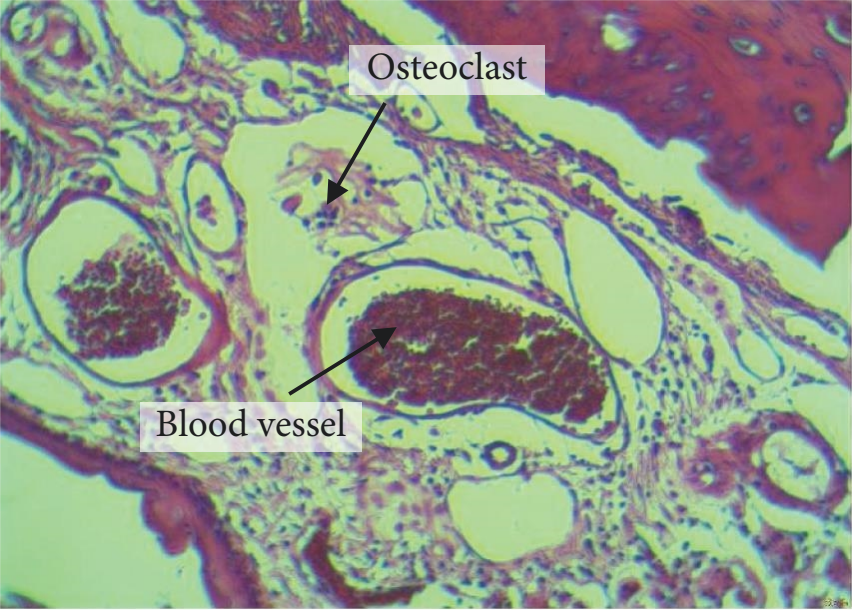
(A)

**Figure:**
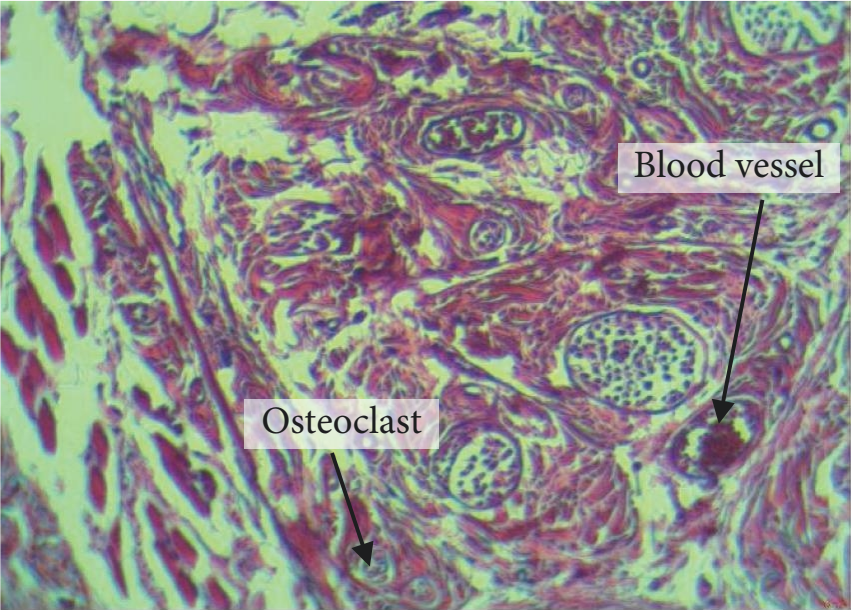
(B)

**Figure:**
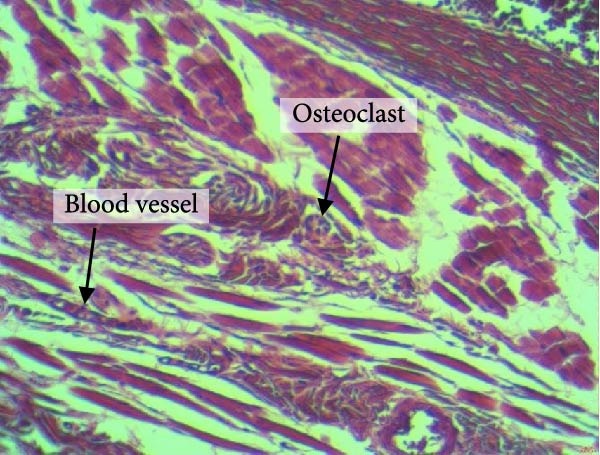
(C)

**Figure:**
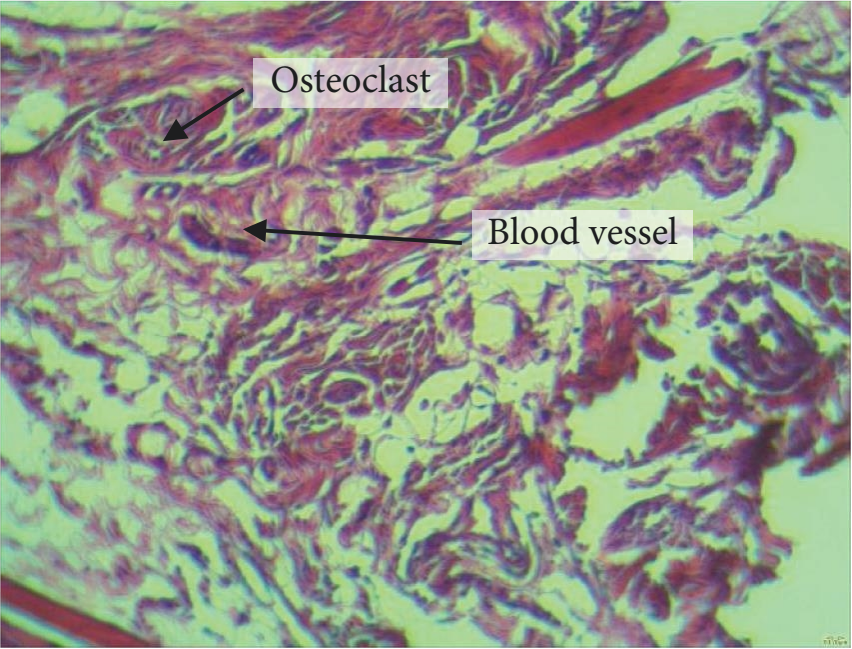
(D)

The results of four‐way ANOVA for the effects of time, stress, orthodontic treatment, and CE injection on the number of Howship’s lacunae, blood vessels, and osteoclasts are presented in Table 2. As shown, time had a significant effect on the number of Howship’s lacunae (*p*  < 0.001) such that the mean number of Howship’s lacunae at 40 days was significantly higher than that at 21 days. Stress had a significant effect on the number of Howship’s lacunae as well (*p*  < 0.001), such that the mean number of Howship’s lacunae was significantly higher in the stressed rats compared with those without stress. Orthodontic treatment had a significant effect on the number of Howship’s lacunae as well (*p*  < 0.001) such that the mean number of Howship’s lacunae in rats subjected to orthodontic treatment was significantly higher than that in the no‐orthodontic treatment subgroups. The CE injection had a significant effect on the number of Howship’s lacunae as well (*p*  < 0.001) such that the mean number of Howship’s lacunae was significantly higher in rats that received CE injections compared with others.

**Table 2:** Results of four‐way ANOVA regarding the effects of time, stress, orthodontic treatment, and CE injection on the number of Howship’s lacunae, blood vessels, and osteoclasts.

Experimental factors		Number of how ship lacunnas (mm^2^)			Number of blood vessels (mm^2^)			Number of osteoclasts (mm^2^)		
		Mean	SD	*p*‐Value	Mean	SD	*p*‐Value	Mean	SD	*p*‐Value^†^
Time	21 days	8.85^a^	5.68	**<0.001**	11.33^a^	2.73	**0.005**	12.15^a^	7.98	**<0.001**
	40 days	10.20^b^	6.17	—	11.78^b^	3.30	—	13.28^b^	8.47	—

Stress	No	8.52^a^	6.31	**<0.001**	10.81^a^	2.69	**<0.001**	11.06^a^	8.45	**<0.001**
	Yes	10.74^b^	5.27	—	12.45^b^	3.19	—	14.69^b^	7.53	—

Orthodontics	No	4.53^a^	2.43	**<0.001**	10.25^a^	3.07	**<0.001**	5.85^a^	3.16	**<0.001**
	Yes	15.53^b^	2.14	—	13.12^b^	2.12	—	20.95^b^	3.51	—

Cannabinoid extract	No	9.39	5.97^b^	**<0.001**	10.04^b^	2.35	**<0.001**	12.16^b^	7.94	**<0.001**
	Cannabinoid	11.27	6.01^c^	—	14.66^c^	1.66	—	15.53^c^	8.71	—
	Saline	7.40	5.17^a^	—	9.44^a^	1.52	—	9.69^a^	6.70	—

*Note:* Significant values are shown in bold. Means with same superscript letters are not significantly different (*p* > 0.05).

Abbreviation: SD, standard deviation.

^†^Four way ANOVA followed by Tukey’s test was used.

Time had a significant effect on the number of blood vessels (*p* = 0.005), such that the mean number of blood vessels at 40 days was significantly higher than that at 21 days. Stress had a significant effect on the number of blood vessels as well (*p*  < 0.001), such that the mean number of blood vessels was significantly higher in the stressed rats compared with others. Orthodontic treatment had a significant effect on the number of blood vessels as well (*p*  < 0.001), such that the mean number of blood vessels in rats subjected to orthodontic treatment was significantly higher than that in other subgroups. CE injection had a significant effect on the number of blood vessels as well (*p*  < 0.001), such that the mean number of blood vessels was significantly higher in rats that received CE injections, compared with others.

Time had a significant effect on the number of osteoclasts (*p*  < 0.001), such that the mean number of osteoclasts at 40 days was significantly higher than that at 21 days. Stress had a significant effect on the number of osteoclasts (*p*  < 0.001), such that the mean number of osteoclasts was significantly higher in the stressed rats compared with others. Orthodontic treatment had a significant effect on the number of osteoclasts (*p*  < 0.001), such that the mean number of osteoclasts was significantly greater in rats subjected to orthodontic treatment, compared with others. CE injection had a significant effect on the number of osteoclasts (*p*  < 0.001), such that the mean number of osteoclasts was significantly higher in rats that received CE injections, compared with others.

### 3.3. Effects of Time, Stress, Orthodontic Treatment, and CE Injection on BV/TV and BMD

The results of four‐way ANOVA regarding the effects of time, stress, orthodontic treatment, and CE injection on the BV/TV ratio and BMD are presented in Table 3. As indicated, time had a significant effect on the BV/TV ratio (*p*  < 0.001), such that the mean BV/TV at 40 days was significantly lower than that at 21 days. Stress had no significant effect on this variable (*p* = 0.061). Orthodontic treatment had a significant effect on the BV/TV ratio (*p*  < 0.001), such that the mean BV/TV ratio in rats that underwent orthodontic treatment was lower than that in others. CE injection had a significant effect on the BV/TV ratio as well (*p*  < 0.001), such that the mean BV/TV ratio was significantly lower in rats subjected to CE injection compared with others.

**Table 3:** Results of four‐way ANOVA regarding the effects of time, stress, orthodontic treatment, and CE injection on the BV/TV ratio and BMD.

Experimental factors		BV/TV (%)			BMD (g.cm^−3^)		
		Mean	SD	*p*‐Value	Mean	SD	*p*‐Value^†^
Time	21 days	26.27^b^	7.19	**<0.001**	2.35^b^	0.47	**<0.001**
	40 days	23.06^a^	7.22	—	2.17^a^	0.60	—

Stress	No	24.95^a^	7.30	0.061	2.32^b^	0.47	**<0.001**
	Yes	24.32^a^	7.47	—	2.19^a^	0.63	—

Orthodontics	No	28.65^b^	7.07	**<0.001**	2.57^b^	0.45	**<0.001**
	Yes	19.88^a^	4.19	—	1.90^a^	0.42	—

Cannabinoid extract	No	27.79^b^	5.68	**<0.001**	2.54^b^	0.46	**<0.001**
	Cannabinoid	17.56^a^	2.20	—	1.76^a^	0.39	—
	Saline	29.97^c^	6.44	—	2.57^b^	0.30	—

*Note:* Significant values are shown in bold. Bone volume fraction = (BV/TV) × 100. Means with same superscript letters are not significantly different (*p* > 0.05).

Abbreviations: BMD, bone mineral density within volume of interest (VOI); BV, bone volume within volume of interest (VOI); SD, standard deviation; TV, total volume of interest (VOI).

^†^Four way ANOVA followed by Tukey’s test was used.

Time had a significant effect on BMD (*p*  < 0.001), such that the mean BMD at 40 days was significantly lower than that at 21 days. Stress had a significant effect on BMD as well (*p*  < 0.001), such that the mean BMD in the stressed rats was significantly lower than that in others. Orthodontic treatment also had a significant impact on BMD (*p*  < 0.001), such that the mean BMD was significantly lower in rats subjected to orthodontic treatment compared with others. Moreover, CE injection had a significant effect on BMD (*p*  < 0.001), such that the mean BMD was significantly lower in rats that received CE injections, compared with others.

## 4. Discussion

Orthodontic force application to the teeth initiates a cascade of biological events to induce OTM. Several factors can affect this process [3, 5]. In recent years, the effects of supplements, vitamins, and different medications on OTM have been the topic of numerous investigations [47–50]. Knowledge about the effects of drugs taken by patients during the course of orthodontic treatment on OTM and the expected duration of treatment is highly important for orthodontists. Evidence shows that administration of some medications, such as methotrexate, in rats under orthodontic treatment was associated with an increase in the number of osteoclasts and blood vessels, and a reduction in the BV/TV ratio and root resorption, accelerating OTM [5]. Another study demonstrated that administration of melatonin in rats during the course of orthodontic treatment enhanced bone formation and prevented bone resorption, decelerating OTM [51]. The present study was the first to assess the simultaneous effects of CE injection and stress in acute and chronic forms on OTM in rats. Cannabinoids have been used for pleasure and therapeutic purposes for thousands of years. The endocannabinoid system plays a significant role in bone metabolism through CB1 and CB2 receptors. CB1 receptors are primarily located in the central nervous system but also influence sympathetic nerve signaling to bone, whereas CB2 receptors are expressed in bone cells and directly regulate osteoclast and osteoblast activity [52]. Activation of CB2 receptors promotes osteoclastogenesis, leading to bone resorption, while CB2 antagonism has been shown to prevent bone loss [23–25]. These findings suggest that cannabinoid administration can accelerate bone remodeling by enhancing osteoclastic activity during OTM.

Studies on cannabinoids and OTM in rats are limited but insightful. Klein et al. [53] examined dronabinol, a synthetic THC, finding it reduced OTM by decreasing bone resorption on the compression side while increasing formation on the tension side, despite more osteoclasts and osteoblasts histologically. This suggests dronabinol inhibits osteoclast activity, slowing movement. The study concluded dronabinol attenuates OTM, with implications for bone recovery processes, potentially extending treatment duration [53]. Evidence shows that in CB2‐deficient rats, the bone tissue had a fewer number of osteoclasts compared with the control group; these results highlight the fact that a shortage of osteoclasts protects against BMD loss [52]. Cannabinoids have been reported to enhance fracture healing by promoting collagen cross‐linking and improving the biomechanical quality of bone [54]. However, clinical studies have shown that chronic cannabis use in humans is associated with decreased BMD and increased fracture risk [28]. This discrepancy highlights the complexity of cannabinoid effects, which may depend on dosage, duration, and species differences. The human body is subjected to stress following exposure to a number of physical and emotional stimuli, and releases glucocorticoids, such as the cortisol hormone, which is released under stressful conditions [55].

Long‐term stress (40 days) resulted in increased plasma corticosterone levels and enhanced tooth movement due to greater osteoclast activity, indicating increased bone resorption [15]. The majority of animal studies on the effects of glucocorticoid injections confirmed that high doses of glucocorticoids can cause osteoporosis and decrease BMD [56]. Bone is a dynamic tissue, which undergoes constant remodeling. The bone resorption and formation processes are influenced by the activity of osteoclasts and osteoblasts. On the other hand, osteoblasts and osteoclasts are influenced by the glucocorticoid and cannabinoid receptors [57]. Nonetheless, some other researchers found that administration of cannabinoids in rats decreased cancellous bone loss and improved the mechanical properties of femoral bone [58]. Another study confirmed the healing effects of cannabinoids on bone, and showed that they can enhance healing of bone fractures in the long term by improving the biomechanical quality of the newly formed bone [54].

The present results indicated higher activity of osteoclasts and greater angiogenesis in rats subjected to orthodontic force application alone, compared with those under no orthodontic treatment; however, the number of Howship’s lacunae, BD/TV ratio, and BMD followed a descending trend in the former rats. On the other hand, the osteoclastic activity and angiogenesis were greater in rats that received 5 mg/kg CE injections compared with others; however, the number of Howship’s lacunae, BD/TV ratio, and BMD followed a descending trend in them, which was in line with previous findings [59–63]. Also, osteoclasts were more active, and angiogenesis increased in rats subjected to acute and chronic stress, but the number of Howship’s lacunae, BD/TV ratio, and BMD decreased in them. Additionally, the rats subjected to orthodontic force application, CE injection, and acute and chronic stress simultaneously showed the greatest increase in the number of osteoclasts and blood vessels and the greatest reduction in the number of Howship’s lacunae, BV/TV ratio, and BMD, showing the highest rate of OTM, compared with others. Nonetheless, several factors may affect OTM measurement, such as deformation of orthodontic appliances during mastication, mesial shift of the second molar, and growth and development of the skull during the course of treatment [40]. It should be noted that all measurements were made by one operator in the present study to prevent the confounding effect of inter‐observer differences on the results.

## 5. Conclusion

Cannabinoids such as marijuana are highly popular. Also, people are subjected to various stressful conditions in their daily life. The effects of cannabinoids on CB2 receptors, OTM, and alveolar bone remodeling have not been well understood and are in need of further investigations.

This study showed that CE injection simultaneous with stress exposure increased OTM and bone remodeling in rats and may serve as a key regulator of osteoclastic activity and angiogenesis and play an important role in bone metabolism. Using a specific breed of rats may disregard genetic variations and limit the generalizability of the findings. Also, generalization of the results of animal studies should be done with caution, and the results should be verified in clinical trials. Daily CE injections might have contributed to stress in rats, potentially confounding the observed effects. Although all animals underwent similar handling to minimize this bias, this factor should be considered when interpreting the findings. Considering the obtained results, orthodontists should take into account the effects of cannabinoids and stressful conditions on OTM. Concerning the existing controversy regarding the effects of cannabinoids on the bone tissue, future studies are required on the effects of different doses of cannabinoids and also on the involved mechanisms in this process.

## Ethics Statement

The Ethics Committee of Kermanshah University of Medical Sciences approved this study (IR.KUMS.REC.1399.120).

## Consent

All authors consented to the publication of this manuscript.

## Disclosure

All authors participated in manuscript revision and approved the final manuscript.

## Conflicts of Interest

The authors declare no conflicts of interest.

## Author Contributions

All authors conceptualized and designed the research, contributed to acquisition, and performed analysis of data, wrote the manuscript, and translated to English.

## Funding

No funding was received for this manuscript.

## Data Availability

The data that support the findings of this study are available from the corresponding author upon reasonable request.
